# Two New Species of *Impatiens* from China, and Taxonomic Insights into the Longifilamenta Group, Which Is Endemic to China

**DOI:** 10.3390/plants10081697

**Published:** 2021-08-18

**Authors:** Yong-Xiu Song, Yan Xiao, Shuai Peng, Yi-Yan Cong, Guang-Wan Hu

**Affiliations:** 1 College of Life Sciences, Hunan Normal University, Changsha 410081, China; SongYongXiu130@163.com (Y.-X.S.); yanxiao_begonia@163.com (Y.X.); 2 CAS Key Laboratory of Plant Germplasm Enhancement and Specialty Agriculture, Wuhan Botanical Garden, Chinese Academy of Sciences, Wuhan 430074, China; pengshuai183@163.com; 3 Shanghai Chenshan Plant Science Research Center, Chinese Academy of Sciences, Shanghai 430074, China; 4 Sino-Africa Joint Research Center, Chinese Academy of Sciences, Wuhan 430074, China; 5 Wuhan Botanical Garden, Chinese Academy of Sciences, University of Chinese Academy of Sciences, Beijing 100049, China

**Keywords:** Balsaminaceae, China, *Impatiens lihengiana*, *Impatiens longshanensis*, morphology, phylogeny, taxonomy

## Abstract

*Impatiens longshanensis* (The LSID for the name *Impatiens longshanensis* is: 77219154-1) sp. nov. and *I. lihengiana* (The LSID for the name *I. lihengiana* is: 77219153-1) sp. nov., from Hunan, China, are described and illustrated here. The molecular phylogenetic study suggests that *I. longshanensis* and *I. lihengiana* should be placed in the *I.* sect. *Impatiens*. A detailed description, diagnostic characters between the two new species and allied species, pollen and seed morphology, and color photographs are provided. In addition, based on wide sampling, we found that the longifilamenta group, an endemic group to China, whose members have basal lobes of lateral united petals with long filamentous hairs, shows significant morphological variability. In this paper, we discuss the taxonomic significance of morphological characteristics within this group. Based on a literature review and observation of living materials in the field, an updated identification key for this group is also proposed.

## 1. Introduction

Balsaminaceae comprises two genera, the monotypic *Hydrocera* Blume ex Wight & Arnott [1] and *Impatiens* Linnaeus [2]. *Impatiens* is one of the most species-rich genera of angiosperms, having more than 1000 species. These are mainly distributed in the mountainous regions of the tropics and subtropics, and have tropical Africa, Madagascar, South India and Sri Lanka, Sino-Himalaya, and South-East Asia as their centers of diversity [3,4]. The genus is distinguished by zygomorphic flowers with tremendous diversity in corolla color and morphology, and lateral petals always united in pairs. The fruit is a fleshy, explosive capsule, and seeds are often dispersed elastically from valves when ripe [5,6]. *Impatiens* species occur in diverse habitats, such as in forest understories, roadside ditches, valleys, abandoned fields, along streams and in seepages, usually in mesic or wet conditions, although some species can tolerate drier habitats [6]. Due to their beautiful appearance and long flowering period, many species of *Impatiens* are grown all over the world as ornamental plants [7]. Many new species of *Impatiens* have been discovered and described in recent years [8,9,10,11,12,13,14,15]. There are more than 280 species of *Impatiens* distributed in China [5,16,17]. Most of these are found in southwestern China, which is a key diversity area of the genus.

As is well known, *Impatiens* is notoriously difficult to classify [3,18]. Morphologically, *Impatiens* are usually fleshy plants, with fine and fragile flowers, usually folded and coalesced in dried specimens, hence losing their original shape and therefore difficult to reconstruct. The botanist Hooker called them “a terror to botanists” and “deceitful above all plants, and desperately wicked”. Yu et al. [6] divided the genus *Impatiens* into two subgenera, *I.* subgen. *Clavicarpa* and subgen. *Impatiens* [15], based on both morphological and molecular evidence, and the latter was further subdivided into seven sections: *Semeiocardium, Racemosae*, *Fasciculatae*, *Impatiens*, *Tuberosae*, *Scorpioidae*, and *Uniflorae.*

The longifilamenta group in *Impatiens* was once treated as *I.* sect. *longifilamenta* [17], then merged into *I.* sect. *Impatiens* [6]. This group is endemic to China, and markedly characterized by its 1- or 2-flowered racemose inflorescences, pedicel with 2 bracts, lower sepal saccate or funnelform, with striate, basal lobes of lateral united petals with a filamentous long hair. However, this group has not attracted much attention, and there are few studies related to it. In this paper, we discuss the morphological characteristics with taxonomic significance in this group, and provide its updated identification key.

During the botanical explorations from 2012 to 2020 in Longshan County, Hunan province, the authors encountered two interesting species of *Impatiens* growing on wet shady habitats under an evergreen forest. Specimens were collected carefully, and the flowers and fruits were preserved in formalin-acetic-alcohol (FAA) solution for further identification. After careful examination of the relevant specimens and literature [5,16,17], the authors confirm the two as new species, and describe them here.

## 2. Results

### 2.1. Taxonomic Treatment of the New Species

#### 2.1.1. Taxonomic Description of *Impatiens longshanensis*

**Figure 1:**
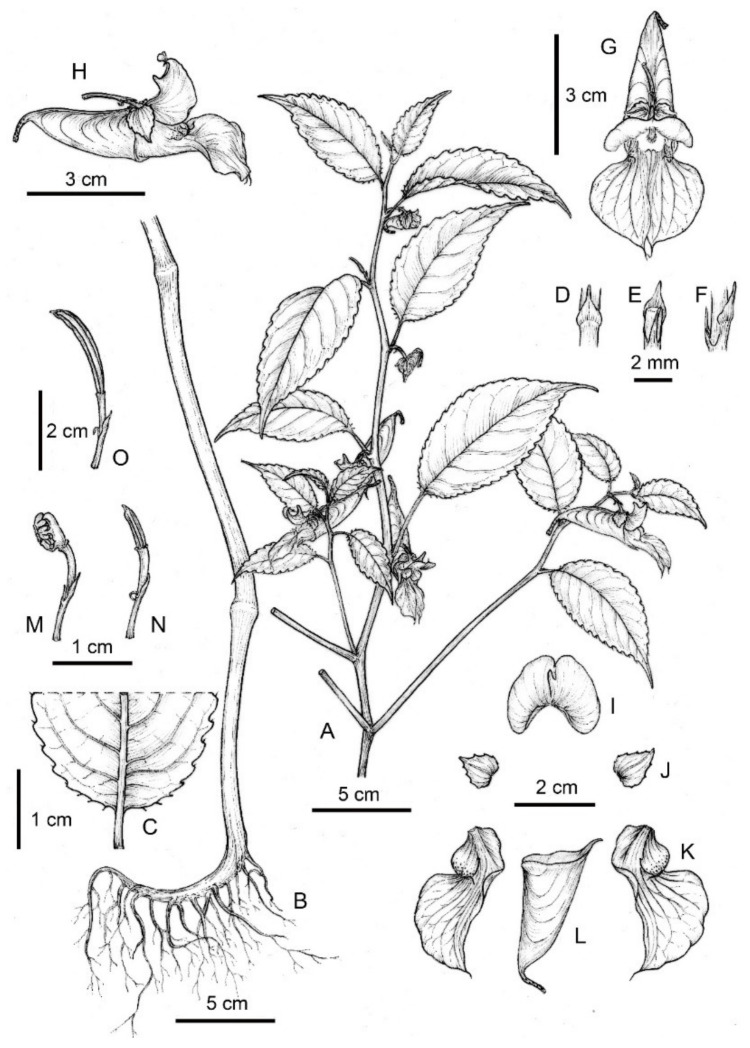
*Impatiens longshanensis*. (**A**) Plant; (**B**) root; (**C**) basal portion of abaxial leaf surface; (**D**) bracts in dorsal view; (**E**) bracts in front view; (**F**) bracts in lateral view; (**G**) flower in anterior view; (**H**) flower in lateral view; (**I**) dorsal petal; (**J**) lateral sepal; (**K**) lateral united petal; (**L**) lower sepal; (**M**) anthers; (**N**) ovary; (**O**) capsule.

**Figure 2:**
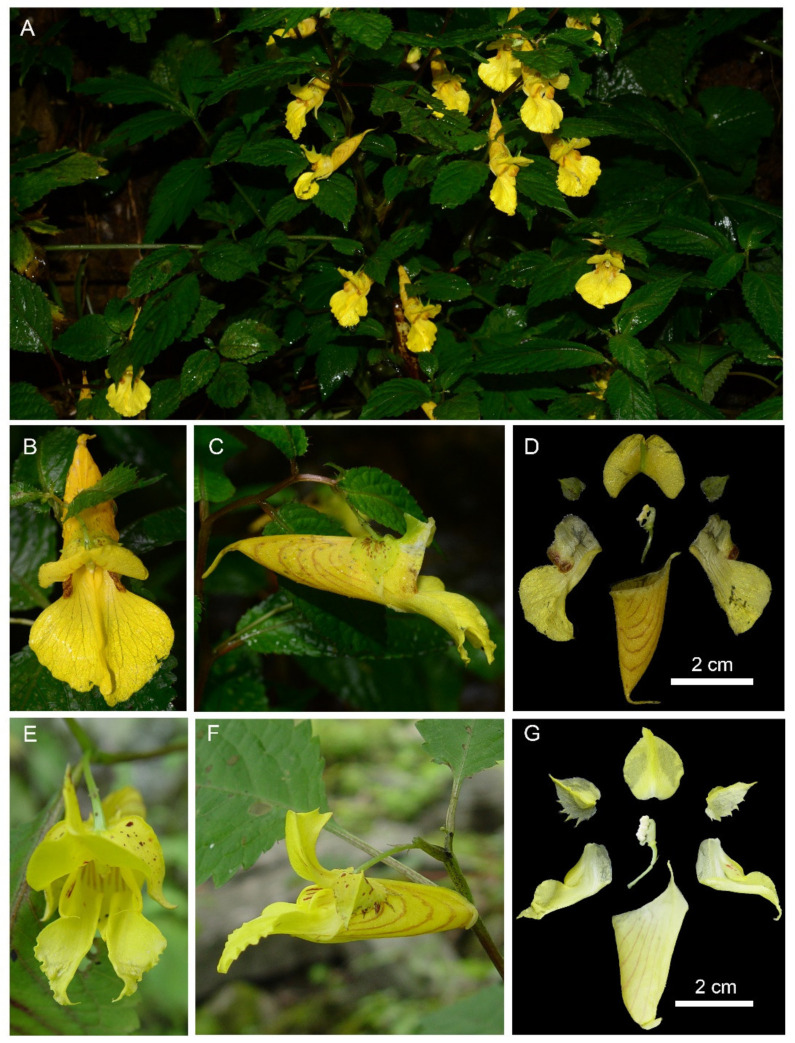
*Impatiens longshanensis*. (**A**) Habit; (**B**) anterior view of flower; (**C**) lateral view of flower; (**D**) flower structure. (**E–G**) *Impatiens dicentra*. (**E**) Anterior view of flower; (**F**) lateral view of flower; (**G**) flower structure.

*Impatiens longshanensis* Y. Y. Cong & Y. X. Song, sp. nov. Figure 1 and Figure 2 and Figure S1.

Type: China, Hunan, Longshan County, Bamian Mountain, Zisheng Bridge, under moist and shady places, 109°15′05.92″ E, 28°52′30.24″ N, altitude 1194 m, 7 October 2020, Yi-Yan Cong 35,443 (holotype: HIB, isotype: HNNU).

##### Diagnosis

*Impatiens longshanensis* is morphologically similar to *I. dicentra* Franch. ex Hook. f., but differs due to its suborbicular lamina base (vs. lamina base cuneate); green, equilateral lateral sepals, 5.5–7 mm wide, coarsely dentate on both sides, and inconspicuously thickened abaxial midvein (vs. yellow, inequilateral, 12–14 mm wide, coarsely dentate on one side, rarely entire, abaxial midvein narrowly carinate); lower sepal 1.85–2.3 cm deep (vs. 3–5 cm deep); reniform dorsal petal (vs. orbicular); oblong basal lobe (vs. lanceolate); and dolabriform distal lobes (vs. lanceolate).

##### Additional Material Examined

China, Hunan, Longshan County, Bamian Mountain, 109°26′37″ E, 28°96′58″ N, altitude 1336 m, 27 July 2013, Yan Xiao LS-2238 (CSH).

##### Description

Annual herb, 45–80 cm tall. Stem erect, slender, slightly ridged base 0.5–0.7 cm in diam, well branched, nodes swollen in lower part. Leaves alternate, petiole 1–3 cm. Lamina 4.5–8 × 1.2–3.9 cm, ovate or narrowly elliptic, membranaceous, glabrous on both surfaces, apex cuspidate, base suborbicular, with 3–4 pairs stipitate glands at basal margin, margin crenate-serrate, setose between marginal teeth, lateral veins 5–8 pairs. Inflorescences in upper leaf axils, 1-flowered; peduncles short, 0.6–1.1 cm long. Pedicels 2-bracteate, lower bracts linear, upper bracts ovate, persistent. Flowers pale yellow, large, 3–4.5 cm long. Sepals: lateral sepals 2, broadly ovate-orbicular, equilateral, 6.5–9 mm long, 5.5–7 mm wide, green, acuminate at apex, coarsely dentate on both sides, abaxial midvein inconspicuously thickened; lower sepal red striate, saccate, 1.85–2.3 cm deep excluding spur, mouth 1.2–1.6 cm wide, anterior gradually narrowed downward into a long beak, base gradually narrowed into a spur, ca 1 cm, incurved, 2-lobed. Petals: dorsal petal reniform, 11–13.5 mm long, 16–22 mm wide, base suborbicular, apex emarginate, shortly rostellate, abaxial midvein cristate, green, lateral united petals not clawed, 1.55–2.1 cm long, 2-lobed, basal lobe 8–12 × 6–8.5 mm, oblong, apex with a filamentous long hair, distal lobes 18–25 × 8.5–13 mm, dolabriform, margin entire, apex obtuse, abruptly narrowed into a short filamentous hair, auricle inflexed. Stamens 5, 5–7.5 mm long, filaments linear, free for about 1/2 of their length. Anthers ovoid, joined into a ring surrounding the ovary apex, apex obtuse, 2–2.5 mm long; ovary superior, 4–5.5 mm long, 5-carpellate, erect, fusiform, placentation axile. Capsule linear, 1.6–2.5 cm long, fleshy, 5-valved. Seeds many, subellipsoid.

##### Etymology

The specific epithet “longshanensis” refers to the locality of the type specimen, Longshan County, Hunan, China.

Phenology: Flowering and fruiting were observed in the field from September to November.

##### Micromorphological Observations

**Figure 3:**
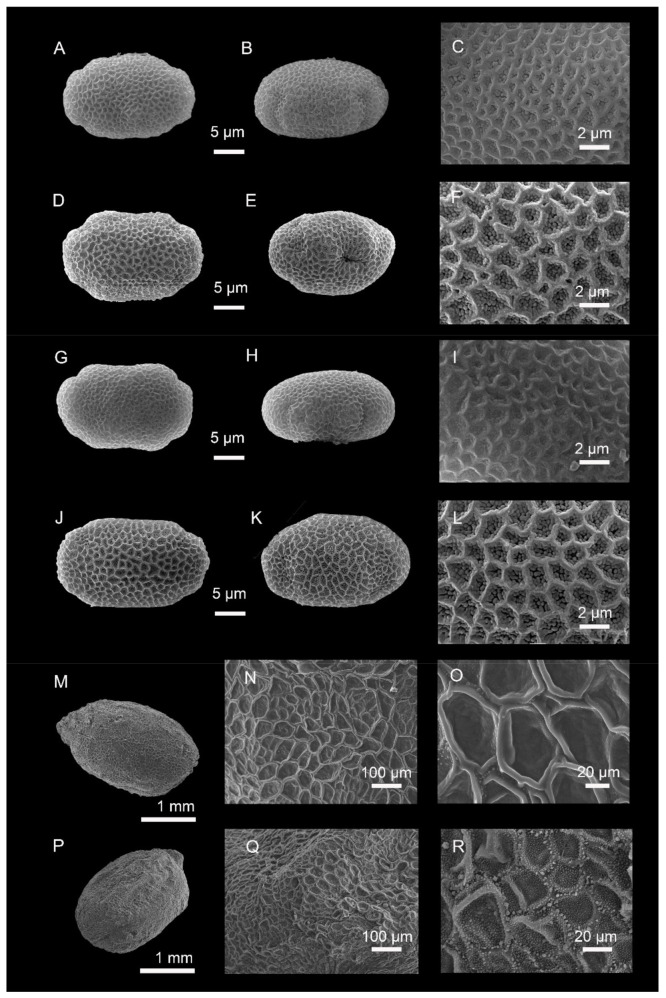
(**A**–**C**,**M**–**O**) *Impatiens longshanensis*; (**D**–**F**,**P**–**R**) *I. dicentra*; (**G**–**I**) *I. lihengiana*; (**J**–**L**) *I. davidii;* (**A**–**L**) scanning electron microscope images of pollen grains; (**A**,**D**,**G**,**J**) polar view; (**B**,**E**,**H**,**K**) equatorial view; (**C**,**F**,**I**,**L**) partial view; (**M**–**R**) scanning electron microscope images of seeds; (**M**,**P**) whole view; (**N**,**O**,**Q**,**R**) partial view.

Pollen grains: *Impatiens longshanensis* and *I. dicentra* are oblong in polar view, 4-colpate, exine with irregularly reticulate ornamentation, dense granules in lumina, the former average size of E1 × E2 = 26.12 (23.83–27.71) × 15.05 (14.37–15.56) μm (Figure 3A–C), the latter average size of E1 × E2 = 29.66 (26.20–32.81) × 18.04 (16.73–20.01) μm (Figure 3D–F). The surface of the pollen grains under SEM of the two species appears to be similar.

Seeds: seeds of *I. longshanensis* subellipsoid, 2.47 × 2.22 mm, ratio of L (length)/W (width) = 1.11, under SEM (Figure 3M–O), surface with irregularly reticulate ornamentation, sparse granules can be seen under high magnification (Figure 3O). Seeds of *I. dicentra* subellipsoid, 2.50 × 2.20 mm, ratio of L (length)/W (width) = 1.14, under SEM (Figure 3P–R), surface with irregularly reticulate ornamentation, dense granules can be seen under high magnification (Figure 3R).

##### Habitat and Distribution

**Figure 4:**
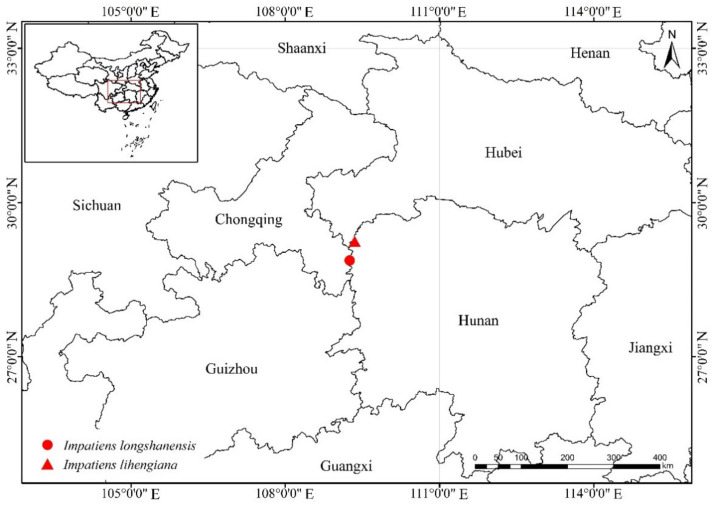
Geographic distribution of *Impatiens longshanensis* and *Impatiens lihengiana* in China.

*Impatiens longshanensis* is currently known only from the type locality in Longshan County, Hunan Province, China (Figure 4). It was found growing in shaded moist places, along creek sides, at altitudes of between 1000 and 1300 m.

#### 2.1.2. Taxonomic Description of *Impatiens lihengiana*

**Figure 5:**
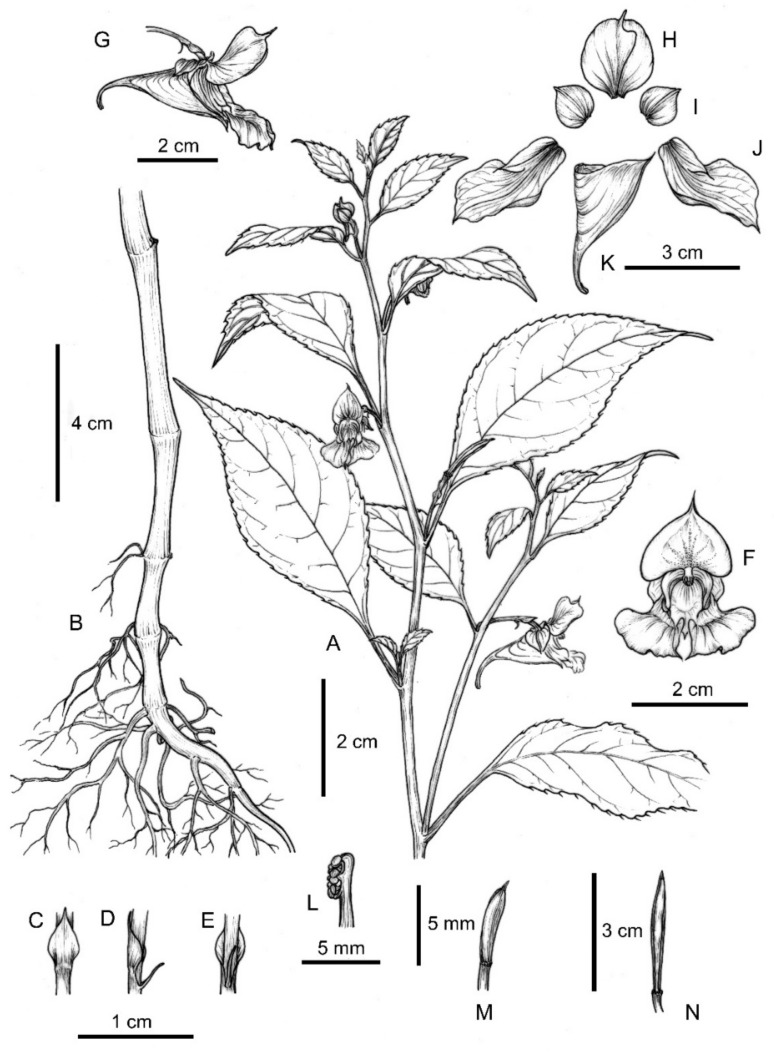
*Impatiens lihengiana* (**A**) Plant; (**B**) root; (**C**) bracts in dorsal view; (**D**) bracts in lateral view; (**E**) bracts in anterior view; (**F**) flower in anterior view; (**G**) flower in lateral view; (**H**) dorsal petal; (**I**) lateral sepal; (**J**) lateral united petal; (**K**) lower sepal; (**L**) anthers; (**M**) ovary; (**N**) capsule.

**Figure 6:**
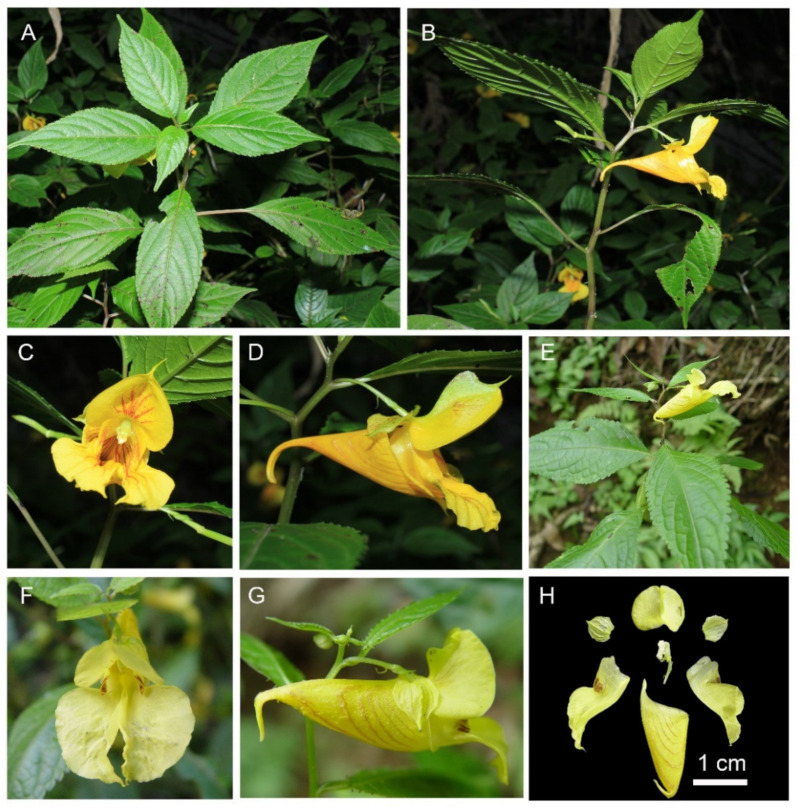
*Impatiens lihengiana*. (**A**) Plant; (**B**) flower branches; (**C**) anterior view of flower; (**D**) lateral view of flower. (**E**–**H**) *Impatiens davidii*. (**E**) Flower branches; (**F**) anterior view of flower; (**G**) lateral view of flower; (**H**) flower structure.

*Impatiens lihengiana* Y. Y. Cong & G. W. Hu, sp. nov. Figure 5 and Figure 6 and Figure S2.

Type: China, Hunan, Longshan County, Huoyanguitang Cave, under moist and shady places, 109°20′59″ E, 29°14′41″ N, altitude 517 m, 4 November 2012, Yan Xiao LS-794 (holotype: CSH, isotype: CSH).

##### Diagnosis

The new species is morphologically similar to *Impatiens davidii* Franch, but differing in having narrowly elliptic or narrowly ovate-elliptic leaf blades (vs. ovate-oblong or ovate-lanceolate); petiole 1.5–2.5 cm (vs. 4–8 cm); lateral sepals yellow-green, purple spotted, 1-veined (vs. yellow, unspotted, 9-veined); lower sepal funnelform (vs. saccate); dorsal petal apex long rostellate (vs. short rostellate); lateral united petals not clawed, 2.5–2.8 cm long (vs. clawed, 1.5–2 cm long); and basal lobe ovate-lanceolate (vs. oblong).

##### Description

Annual herb, 60–75 cm tall. Stem erect, slender, base 0.5–0.7 cm in diam, rarely branched. Leaves alternate, petiole 1.5–2.5 cm. Lamina 6–12.5 × 3–4.2 cm, narrowly elliptic or narrowly ovate-elliptic, membranaceous, glabrous on both surfaces, apex cuspidate, base cuneate, margin crenate-serrate, setose between marginal teeth, lateral veins 6–8 pairs. Inflorescences in upper leaf axils, 1-flowered; peduncles 0.6–1.1 cm long. Pedicels 2-bracteate, lower bracts linear, upper bracts ovate ca. 0.5 cm long, ca. 0.3 cm wide, apex long acuminate. Flowers yellow, large, 4–4.5 cm long. Sepals: lateral sepals 2, yellow-green, purple spotted, suborbicular, 8–10 mm long, ca. 8 mm wide, 1-veined, acuminate at apex, lower sepal red striate, funnelform, 2–2.2 cm deep excluding spur, mouth 18–22 mm wide, base gradually narrowed into a spur, 10–12 mm long, incurved, 2-lobed. Petals: dorsal petal suborbicular, 15–17 mm long, 12–14 mm wide, apex long rostellate, abaxial midvein cristate, green, lateral united petals not clawed, 2.5–2.8 cm long, 2-lobed, basal lobe 10–12.5 × 2–3.5 mm, ovate-lanceolate, apex with a filamentous long hair, ca. 1 mm long, distal lobes 19–22 × 10–12 mm, dolabriform, apex obtuse, constricted into a filamentous hair, auricle inflexed. Stamens 5, ca. 5 mm long, filaments linear, free for about 1/2 of their length. Anthers ovoid, joined into a ring surrounding the ovary apex, apex obtuse, ca. 4.5 mm long; ovary fusiform, superior, ca. 5 mm, 5-carpellate, erect, placentation axile. Capsule linear, 3.3–3.5 cm long, fleshy, 5-valved.

##### Etymology

The specific epithet “lihengiana” is given in honor of Prof. Heng Li, a taxonomist at Kunming Institute of Botany, Chinese Academy of Sciences, who has made significant contributions to plant taxonomy.

Phenology: Flowering and fruiting were observed in the field from September to November.

##### Habitat and Distribution

*Impatiens lihengiana* is currently known only from the type locality in Longshan County, Hunan Province, China (Figure 4). It grows in shaded moist places, along streams, between 450 and 650 m.

##### Micromorphological Observations

Pollen grains: *Impatiens lihengiana* and *I. davidii* are oblong in polar view, 4-colpate, exine with irregularly reticulate ornamentation, dense granules in lumina, the former average size of E1 × E2 = 25.53 (22.28–28.58) × 15.28 (13.15–16.92) μm (Figure 3G–I), the latter average size of E1 × E2 = 25.10 (22.68–27.84) × 16.76 (15.12–18.01) μm (Figure 3J–L). The surface of the pollen grains under SEM of the two species appears to be similar.

### 2.2. Molecular Phylogenetic Analysis

**Figure 7:**
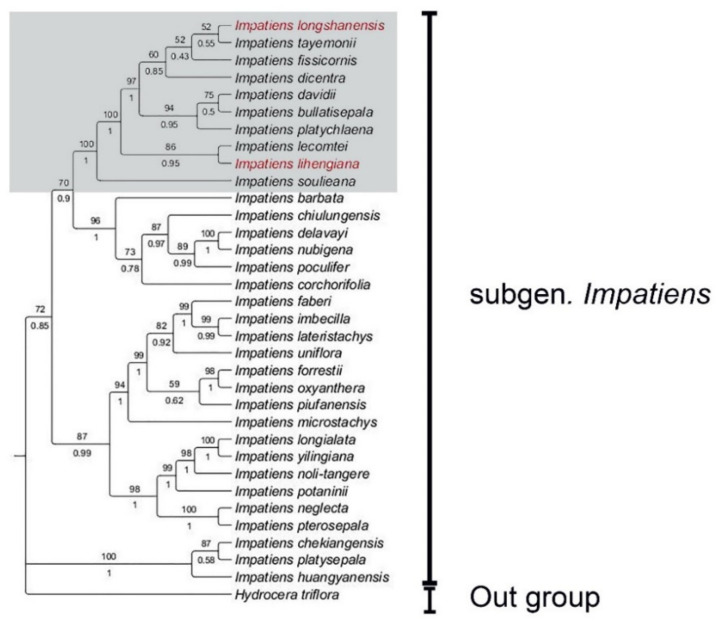
Phylogenetic tree inferred from Bayesian analysis and maximum likelihood analyses based on a combined dataset of nrDNA ITS and cpDNA *atpB-rbcL* sequences. Values above the branches are maximum parsimony bootstrap supports, and below the branches are Bayesian posterior probabilities. Two new species are highlighted in red, longifilamenta group is highlighted in gray background.

Consistent with Yu et al. [6], *Impatiens* can be divided into *I.* subgen. *Clavicarpa* and *I.* subgen. *Impatiens*. Within *I.* subgen. *Impatiens*, several sections can be recognized. The molecular phylogenetic analysis of *Impatiens* based on ITS and *atpB-rbcL* supported ten species, namely, *I. soulieana*, *I. lecomtei*, *I. platychlaena*, *I. bullatisepala*, *I. davidii*, *I. dicentra*, *I. fissicornis*, *I. tayemonii*, and our two proposed new species, to cluster into a clade which belongs to *I.* sect. *Impatiens* (Figure 7). This result is also consistent with the morphological classification in which these species are all characterized by lateral united petals with a long filamentous appendage.

### 2.3. Taxonomic Insights

The results of our phylogenetic reconstruction show that the longifilamenta group in *Impatiens* is a phylogenetically and morphologically cohesive group (Figure 7). As is currently known, this group consists of 25 species growing as annual herbs, characterized by 1–3 flowered racemose inflorescence, and basal lobes of lateral united petals having an apex with a filamentous appendage [5,13,14,19,20,21,22]. The morphological characteristics of lateral sepals, lower sepals, and lateral united petals have a taxonomic significance within these species, crucial for their identification. They are endemic to China, distributed from Taiwan to Qinghai-Tibet Plateau. 

The molecular data supported that *Impatiens soulieana* is a separate subclade (Figure 7). Morphologically, the apex of distal lobes in *Impatiens soulieana* is retuse (a distinctive characteristic that defines this species), whereas the apex of distal lobes in other species in this clade is entire, and the apex is constricted into filamentous hair. Thus, the phylogenetic hypothesis is congruent with the observed morphological distinctiveness. Interestingly, the apex of distal lobes in *Impatiens oblongipetala* is also retuse, but more phylogenetic evidence is needed to clearly understand its taxonomic status. 

**Figure 8:**
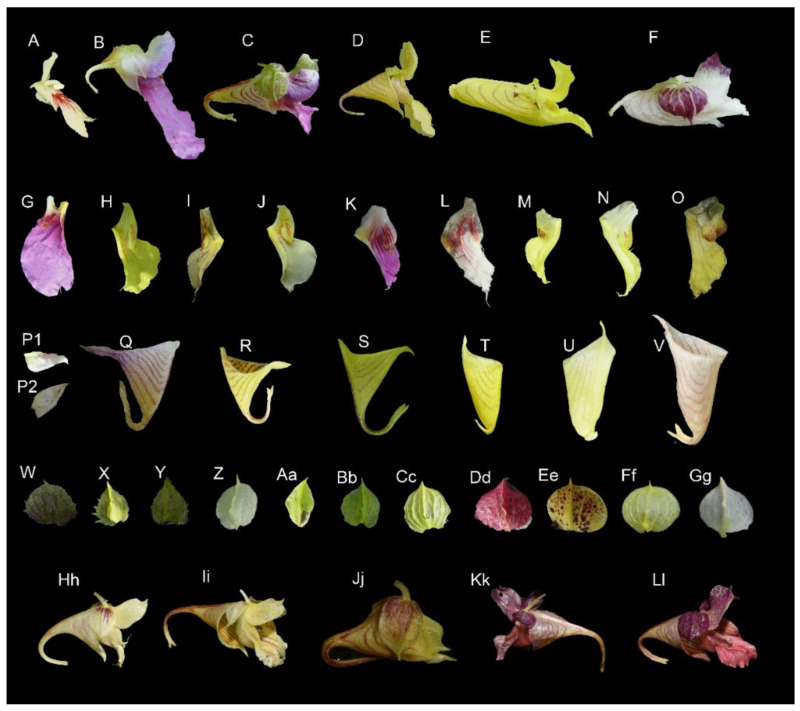
Floral structural diversity of longifilamenta group. (**A–F**,**Hh–Ll**) Flower in lateral view; (**G–O**) lateral united petals; (**P1,P2–V**) lower sepal; (**W–Z**,**Aa–Gg**) lateral sepals; (**A**,**P1,P2**) *I. shennongensis*; (**B**,**G**) *I. oblongipetala*; (**C**,**K**,**Q**,**W**) *I. weihsiensis*; (**D**,**H**,**S**,**Bb**) *I. soulieana*; (**E**,**N**,**U**,**X**) *I. dicentra*; (**F**,**L**,**V**,**Dd–Gg**) *I. platychlaena*; (**I**,**Z**) *I. conaensis*; (**J**,**R**,**Aa**,**Hh–Ll**) *I. toxophore*; (**M**,**T**,**Cc**) *I. davidii*; (**O**,**Y**) *I. longshanensis*.

Based on morphological characteristics, there are significant variations in the structure of the flower within this group (Figure 8A–F). The distinctive characteristic of this group is that the apex of distal lobes of lateral united petals has a filamentous appendage, with few exceptions, and the filamentous appendages are also diverse (Figure 8G–O). Previous descriptions of this group were mostly based on dried herbarium specimens. However, the filamentous appendages of distal lobes also need to be observed with a stereomicroscope and recorded in the field, because their complex morphological characters are often poorly preserved in herbarium materials and, hence, the filamentous appendages may be ignored.

Based on many years of field observations, in addition to literature consultation, the following identification key is proposed, and hence the description herein.

### 2.4. Analytical Key to the Taxa of Longifilamenta Group of Impatiens in China

- 1a Basal lobes of lateral united petals with a filamentous long hair, distal lobes of lateral united petals apex retuse with aseta …………………………………………… 2
- 1b Basal lobes of lateral united petals with a filamentous long hair, distal lobes of lateral united petals apex entire with a filamentous long hair or a seta ………………………………………………………………………………………… 3
- 2a Flowers pale rose pink; lateral sepals abaxial midvein narrowly thickened; distal lobes of lateral united petals oblong or suboblong …………………… *I. oblongipetala*
- 2b Flowers yellow; lateral sepals abaxial midvein narrowly carinate; distal lobes of lateral united petals dolabriform …………………………………………… *I. soulieana*
- 3a Lower sepal funnelform or navicular ……………………………………………… 4
- 3b Lower sepal saccate ………………………………………………………………… 13
- 4a Lower sepal navicular, spur absent ………………………………… *I. shennongensis*
- 4b Lower sepal funnelform, spur 2-lobed ……………………………………………… 5
- 5a Lateral sepals margin 4-or 5-denticulate on one side ………………… *I. weihsiensis*
- 5b Lateral sepals margin entire ………………………………………………………… 6
- 6a Lateral united petals clawed ………………………………………………………… 7
- 6b Lateral united petals not clawed …………………………………………………… 8
- 7a Lateral sepals broadly ovate-cordate, abaxial midvein cristate ……… *I. toxophora*
- 7b Lateral sepals ovate-orbicular, abaxial midvein without cristate …… *I. tayemonii*
- 8a Lateral sepals abaxial midvein carinate narrowly cristate or with a spinelike appendage …………………………………………………………………………………… 9
- 8b Lateral sepals abaxial midvein without carinate ………………………………… 12
- 9a Flowers pink ………………………………………………………………… *I. lecomtei*
- 9b Flowers yellow ……………………………………………………………………… 10
- 10a lateral sepals ovate, abaxial midvein a spinelike appendage; dorsal petal reniform ………………………………………………………………………… *I. cornutisepala*
- 10b lateral sepals orbicular, abaxial midvein carinate or narrowly cristate; dorsal petal orbicular ……………………………………………………………………………11
- 11a Bracts subulate; Lateral sepals 10–20 mm in diam, abaxial midvein acutely carinate ……………………………………………………………………………… *I. brevipes*
- 11b Bracts ovate; Lateral sepals ca. 8 mm in diam, abaxial midvein narrowly cristate ……………………………………………………………………………… *I. mussotii*
- 12a Flowers yellow; lateral sepals suborbicular, 1-veined ……………… *I. lihengiana*
- 12b Flowers pale purple or purple-red; Lateral sepals ovate-orbicular,7-veined …………………………………………………………………… *I. gongchengensis*
- 13a Lateral sepals margin dentate …………………………………………………… 14
- 13b Lateral sepals margin entire ……………………………………………………… 18
- 14a Lateral sepals inequilateral, coarsely dentate on one side ……………… *I. dicentra*
- 14b Lateral sepals equilateral, dentate on both side ………………………………… 15
- 15a Lateral sepals margin irregularly fimbriate-lacerate …………………………… 16
- 15b Lateral sepals margin coarsely dentate …………………………………………… 17
- 16a Flowers yellow, to 4.5 cm deep; lateral sepals margin and abaxial midvein irregularly fimbriate-lacerate; dorsal petal orbicular …………………………… *I. lacinulifera*
- 16b Flowers pale purple, 2–3 cm deep; lateral sepals margin irregularly lacerate; dorsal petal broadly reniform …………………………………………………… *I. platyceras*
- 17a Lateral united petals clawed; lower sepal with a hooked spur; dorsal petal suborbicular ……………………………………………………………………… *I. fissicornis*
- 17b Lateral united petals not clawed; lower sepal with a incurved spur; dorsal petal reniform …………………………………………………………………… *I. longshanensis*
- 18a Lateral united petals clawed ……………………………………………………… 19
- 18b Lateral united petals not clawed …………………………………………………… 22
- 19a Dorsal petal orbicular or suborbicular …………………………………………… 20
- 19b Dorsal petal broadly ovate ………………………………………………………… 21
- 20a Lateral sepals yellow, broadly ovate, 9-veined ………………………… *I. davidii*
- 20b Lateral sepals pale green, orbicular, abaxial midvein with a small sac at base ………………………………………………………………………………… *I. vittata*
- 21a Lateral sepals abaxially plicated; basal lobes of lateral united petals oblong ………………………………………………………………………… *I. plicatisepala*
- 21b Lateral sepals lateral veins reticulate and sunk on abaxial surface with bullate projections among veins; basal lobes of lateral united petals ovate to elliptic …………………………………………………………………………… *I. bullatisepala*
- 22a Lateral sepals abaxial midvein not thickened, many veined ……… *I. platychlaena*
- 22b Lateral sepals abaxial midvein fine or slightly thickened, carinate …………… 23
- 23a Lateral sepals apex aristate-acuminate ……………………………… *I. waldheimiana*
- 23b Lateral sepals apex without aristate-acuminate ………………………………… 24
- 24a Flowers ca. 4 cm deep; lateral sepals abaxial midvein fine, turgid; basal lobes of lateral united petals oblate ……………………………………………………… *I. robusta*
- 24b Flowers 2.5–3 cm deep; lateral sepals abaxial midvein slightly thickened, carinate; basal lobes of lateral united petals ovate-lanceolate ………………………… *I. conaensis*

## 3. Discussion

Based on morphological evidence and molecular phylogenetic study, the two new species should be placed in *Impatiens* sect. *Impatiens*. *I. longshanensis* is similar to *I. dicentra* in its single-flowered inflorescence, yellow flowers, and saccate lower sepal. However, *I. longshanensis* can easily be distinguished from similar species by its green equilateral lateral sepals, reniform dorsal petal, oblong basal lobe, and dolabriform distal lobes. A more detailed morphological comparison between *I. longshanensis* and *I. dicentra* is provided in Table 1. *I. lihengiana* is superficially similar to *I. davidii* in having single-flowered inflorescence, dolabriform distal lobes, and 4-colpate pollen grains, but differs by its yellow-green lateral sepals, purple spotted, 1-veined, funnelform lower sepal, not clawed lateral united petals, and ovate-lanceolate basal lobe. A detailed comparison of similar species is given in Table 2.

Overall, the evidence from the combinations of morphology and phylogeny shows that the longifilamenta group is a cohesive group. For this group, an apex with a filamentous appendage in basal lobes of lateral united petals is the most noticeable feature distinguishing it from other species of *Impatiens*. In addition, this group is endemic to China, and mainly distributed in western Sichuan, Hubei, southern Henan, Guizhou, northwest Guangxi, and Jiangxi, Taiwan, and it favors montane elevations (1000–3000 m). 

Based on the material examined in the present study, the morphological characters of this particular group show a significant variability. The morphology of the lower sepal and lateral sepals shows the diversity of flower structures. The shape of the lower sepal is variable, ranging from navicular, infundibular to saccate (Figure 8P–V). The margin of the lateral sepals is dentate or entire. The abaxial midvein of the lateral sepals ranges from slightly thickened, carinate to a spinelike appendage (Figure 8W–Z,Aa–Gg). Furthermore, the color of flowers of some populations displays a significant variability and instability within intraspecies in these *Impatiens* taxa. For example, in the previous literature [7], lateral sepals of *Impatiens platychlaena* are purple, but in this study, the color changes gradually, ranging from purple, yellow with purple spots to pure yellow, based on years of field observation (Figure 8Dd–Gg). Similarly, the flower of *Impatiens toxophora* is multicolor with the flower color varying from white, yellow to purple within the same population (Figure 8Hh–Ll). These variabilities may blur the lines between species to some extent, hence posing serious challenges to species identification and classification. Therefore, their taxonomic status needs future investigation and more phylogenetic evidence to enhance the understanding of the species relationships within this particular group.

## 4. Materials and Methods

### 4.1. Gross Traits

Specimens of the two new species were collected from Longshan County and quality color photographs were taken in the field. The morphological characteristics of the two species reported in this paper are mostly based on measurements and observations of living plants, specimens, and color photographs.

### 4.2. SEM Observations

Mature whole pollen grains and seeds were collected from flowers and fruits, respectively, in the natural habitats of the plants, and later observed directly under an anatomical macroscope (Olympus SZX10). Dried pollen grains and seeds were carefully mounted on circular metal stubs using double-sided adhesive tape, sputter coated with gold using the JEC-3200 Auto Fine Coater, and then examined and photographed using the JSM-IT500 SEM. Pollen characters were described according to the literature [23,24], and seed characters were described according to the literature [25,26,27].

### 4.3. DNA Sequencing and Phylogenetic Analyses

Genomic DNA of the two new species was extracted from silica gel-dried leaves (Yi-Yan Cong 35443, Yan Xiao LS-794 at HNNU, CSH) using Mag-MK Plant Genomic DNA extraction kits (Sangon Biotech, Shanghai, China). PCR product sequencing was carried out using TSINGKE Biological Technology. Thirty-three representative species from *Impatiens* were chosen to construct a phylogenetic tree with *Hydrocera trifloral* as outgroups. DNA sequences of these 34 species were downloaded from GenBank, with the exception of the two new species, *I. dicentra*, *I. platychlaena*, and *I. longialata.* Species names and GenBank accession numbers are provided in Supplementary Materials Table S1.

For phylogenetic analysis, two molecular markers were used: ITS and *atpB-rbcL* [28,29]. Phylogenetic analysis was conducted using PhyloSuite ver. 1.1.16 [30]. Sequences were assembled and edited in the Mega ver. 7.0.26 [31]. Sequence alignments were carried out using MAFFT ver. 7.222 [32]. The best-fit DNA substitution models were selected by ModelFinder [33]. The Bayesian inference phylogeny was reconstructed using MrBayes 3.2.6 [34] under the GTR + I + G + F model (2 parallel runs, 10 million generations, and sampled every 1000 generations), in which the initial 25% of sampled data was discarded as burn-in. Maximum likelihood phylogenies were inferred using IQ-TREE [35] under the GTR + R3 + F model for 1000 ultrafast [36] bootstraps, in addition to the Shimodaira–Hasegawa-like approximate likelihood-ratio test [37].

## Figures and Tables

**Table 1:** Comparative morphology of *Impatiens longshanensis* and *I. dicentra*.

Characters	*Impatiens longshanensis*	*Impatiens dicentra*
Plant height (cm)	40–80	60–90
Length of petiole (cm)	1–3	2–5
Lateral sepal shape	broadly ovate-orbicular, equilateral	broadly ovate-orbicular, inequilateral
Lateral sepal margin	coarsely dentate on both sides	coarsely dentate on one side, rarely entire
Lateral sepal dorsum	abaxial midvein inconspicuously thickened	abaxial midvein narrowly carinate
Lateral sepal color	green	yellow
Deep of lower sepal (cm)	1.85–2.3	3–5
Length of spur (cm)	ca.1	vix 1
Dorsal petal	reniform	orbicular
Length of lateral united petals (cm)	2.2–2.9	2
Basal lobes	oblong	lanceolate
Distal lobes	dolabriform	lanceolate

**Table 2:** Comparative morphology of *Impatiens lihengiana*, *I. davidii*, and *I. platychlaena*.

Characters	*Impatiens lihengiana*	*Impatiens davidii*	*Impatiens platychlaena*
Plant height (cm)	60–70	ca. 90	60–100
Leaf shape	narrowly elliptic or narrowly ovate-elliptic	ovate-oblong, or ovate-lanceolate	ovate-oblong, ovate, or ovate-lanceolate
Length of petiole (cm)	1.5–2.5	4–8	2–5
Flower color	yellow	yellowish	bicolored: purple and yellow
Lateral sepal shape	suborbicular, purple spotted	broadly ovate	broadly orbicular, purple spotted
Lateral sepal dorsum	1-veined	9-veined	many veined
Lateral sepal color	yellow-green	yellow	brown to purple-red when dry
Lower sepal	funnelform, base gradually narrowed into an incurved spur, 10–12 mm long	saccate, abruptly narrowed into a hooked spur, ca. 8 mm long	deeply saccate, abruptly narrowed into an incurved spur, ca. 6 mm long
Dorsal petal apex	long rostellate	emarginate, shortly rostellate	retuse
Lateral united petals	not clawed, 2.5–2.8 cm	clawed,1.5–2 cm	not clawed, 2.5–3 cm
Basal lobes	ovate-lanceolate, apex with a filamentous long hair	oblong, apex acuminate or caudate	orbicular, apex with a filamentous long hair
Distal lobes	dolabriform, apex obtuse, constricted into a filamentous hair	dolabriform, apex obtuse	dolabriform, longer, with a long filamentous hair
Capsule	linear	linear-cylindric	linear

## Data Availability

Not applicable.

## Supplementary Materials

The following are available online at https://www.mdpi.com/article/10.3390/plants10081697/s1, Figure S1: Image of *Impatiens longshanensis* holotype specimen, Figure S2: Image of *Impatiens lihengiana* holotype specimen, Table S1: GenBank accession numbers for species in the phylogenetic analysis.
