# Supplementary material for: Two New Species of Impatiens from China, and Taxonomic Insights into the Longifilamenta Group, Which Is Endemic to China
Source: Plants (Basel). 2021 Aug 18;10(8):1697. doi: 10.3390/plants10081697 (PMC8398093; doi:10.3390/plants10081697)
Supplement: Supplementary file 1 [file plants-10-01697-s001.zip › plants-1346311-supplementary.pdf]

Supplement Materials.

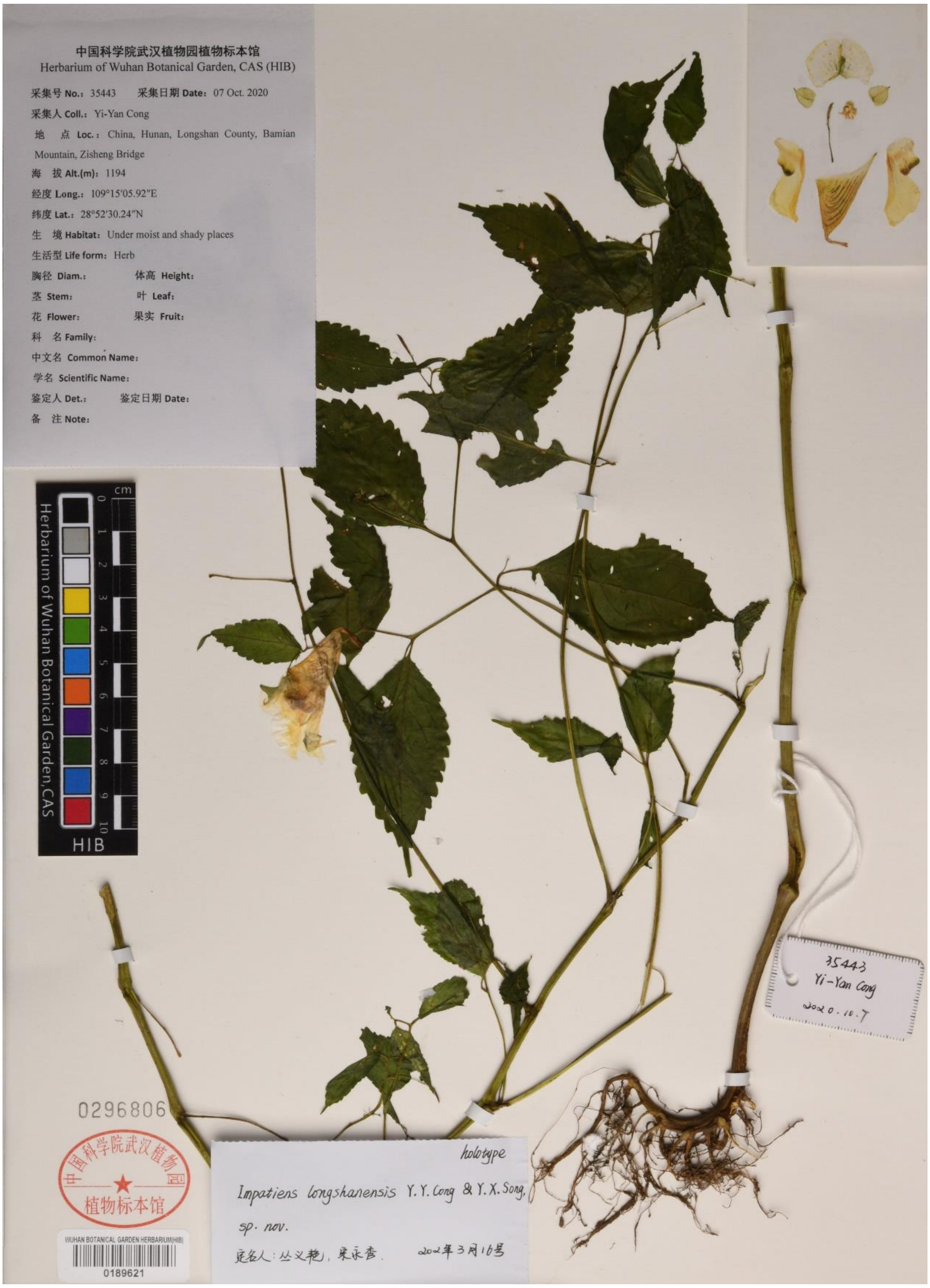

Figure S1. Image of *Impatiens longshanensis* holotype specimen.

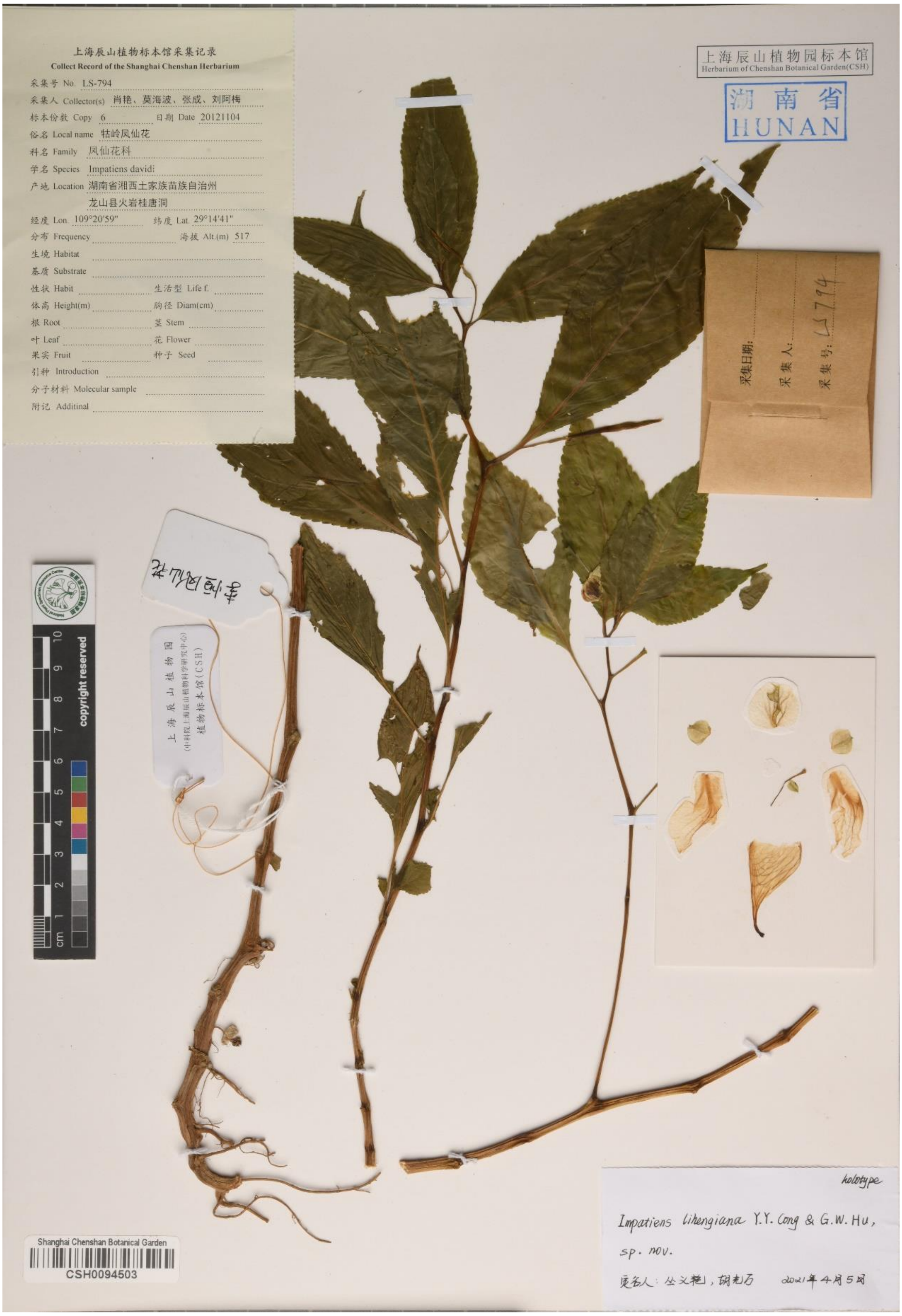

Figure S2. Image of *Impatiens lihengiana* holotype specimen.

Table S1. GenBank accession numbers for species in the phylogenetic analysis.

Species	ITS	atpB-rbcL
Hydrocera triflora	AY348853	DQ147895
Impatiens barbata	AY348750	DQ147818
Impatiens bullatisepala	MZ087896	
Impatiens chekiangensis	KP776064	MN974547
Impatiens chiulungensis	KP776066	KP776016
Impatiens corchorifolia	AY348767	DQ147831
Impatiens davidii	KP776070	MN974551
Impatiens delavayi	AY348773	DQ147836
Impatiens faberi	AY348778	DQ147841
Impatiens fissicornis	MH710828	DQ147844
Impatiens forrestii	AY348784	DQ147847
Impatiens huangyanensis	KP768419	
Impatiens imbecilla	AY348796	DQ147851
Impatiens lateristachys	KP776078	KP776030
Impatiens lecomtei	AY348802	DQ147855
Impatiens microstachys	KP776085	
Impatiens neglecta	MN974569	KP776038
Impatiens noli-tangere	MH710658	DQ147863
Impatiens nubigena	KP776089	KP776040
Impatiens oxyanthera	AY348814	DQ147865
Impatiens piufanensis	KP776094	
Impatiens platysepala	MN974571	MN974555
Impatiens poculifer	MH117575	DQ147870
Impatiens potaninii	MH808399	
Impatiens pterosepala	KP776097	KP776046
Impatiens soulieana	AY348833	DQ147880
Impatiens tayemonii	AY348839	
Impatiens uniflora	AY348846	DQ147888
Impatiens yilingiana	MN974566	MN974549
